# Total and regional bone mineral and tissue composition in female adolescent athletes: comparison between volleyball players and swimmers

**DOI:** 10.1186/s12887-018-1182-z

**Published:** 2018-07-03

**Authors:** João Valente-dos-Santos, Óscar M. Tavares, João P. Duarte, Paulo M. Sousa-e-Silva, Luís M. Rama, José M. Casanova, Carlos A. Fontes-Ribeiro, Elisa A. Marques, Daniel Courteix, Enio R. V. Ronque, Edilson S. Cyrino, Jorge Conde, Manuel J. Coelho-e-Silva

**Affiliations:** 1 CIDAF (UID/DTP/04213/2016), University of Coimbra, Coimbra, Portugal; 2 Portuguese Foundation for Science and Technology (SFRH/BPD/100470/2014), Lisbon, Portugal; 3 Institute for Biomedical Imaging and Life Sciences (IBILI), Faculty of Medicine, University of Coimbra, Coimbra, Portugal; 4 Faculty of Physical Education and Sport, Lusófona University of Humanities and Technologies, Lisbon, Portugal; 5 Department of Medical Imaging and Radiation Therapy, School of Health and Technology, Polytechnical Institute of Coimbra, Coimbra, Portugal; 6 Faculty of Sports Sciences and Physical Education, University of Coimbra, Coimbra, Portugal; 7 Portuguese Foundation for Science and Technology (SFRH/BD/101083/2014), Lisbon, Portugal; 8 Faculty of Medicine, University of Coimbra, Coimbra, Portugal; 9 Laboratory of Pharmacology and Experimental Therapeutics, Institute for Biomedical Imaging and Life Sciences (IBILI), Faculty of Medicine, University of Coimbra, Coimbra, Portugal; 10 Center for Neuroscience and Cell Biology (CNC), Institute for Biomedical Imaging and Life Sciences (IBILI), Faculty of Medicine, University of Coimbra, Coimbra, Portugal; 11 Research Center in Sports Sciences, Health Sciences and Human Development (CIDESD), University Institute of Maia (ISMAI), Maia, Portugal; 12 Laboratory of Metabolic Adaptations to Exercise in Physiological and Pathological conditions (AME2P), Université Clermont Auvergne, Clermont-Ferrand, France; 13 School of Exercise Science, Faculty of Health, Australian Catholic University, East Melbourne, Victoria Australia; 14 Metabolism, Nutrition, and Exercise Laboratory (GEPEMENE), State University of Londrina (UEL), Londrina, Brazil; 15 School of Health and Technology, Polytechnical Institute of Coimbra, Coimbra, Portugal

**Keywords:** DXA, Impact and non-impact loading sports, Exercise, Bone health, Body composition

## Abstract

**Background:**

Exploring the osteogenic effect of different bone-loading sports is particular relevant to understand the interaction between skeletal muscle and bone health during growth. This study aimed to compare total and regional bone and soft-tissue composition between female adolescent swimmers (*n*=20, 15.71±0.93 years) and volleyball players (*n*=26, 16.20±0.77 years).

**Methods:**

Dietary intake was obtained using food frequency questionnaires. Body size was given by stature, sitting height, and body mass. Six skinfolds were measured. Bone mineral content (BMC) and density (BMD), lean soft tissue, and fat tissue were assessed using dual-energy X-ray absorptiometry. Pearson’s product moment correlation coefficients were calculated to examine the relationships among variables, by type of sport. Comparisons between swimmers and volleyball players were performed using student t-tests for independent samples and multivariate analysis of covariance (controlling for age, training history and body size).

**Results:**

Swimmers (BMC: 2328±338 g) and volleyball players (BMC: 2656±470 g) exceeded respectively by 2.1 and 2.8 standard deviation scores the average of international standards for whole body BMC of healthy adolescents. Years of training in swimmers were positively related to the upper limbs BMC (*r*=+0.49, *p*<0.05). In volleyball players, years of training correlated significantly with lower limbs BMD (*r*=+0.43, *p*<0.05). After adjustments for potential confounders, moderate differences (ES-*r*=0.32) between swimmers and volleyball players were noted in BMD at the lower limbs (volleyball players: +0.098 g∙cm^-2^, +7.8%).

**Conclusions:**

Youth female athletes who participate in high-intensity weight-loading activities such as volleyball exhibit moderately higher levels of BMD at the lower limbs compared to non-loading sports such as swimming.

**Electronic supplementary material:**

The online version of this article (10.1186/s12887-018-1182-z) contains supplementary material, which is available to authorized users.

## Background

As life expectancy continues to rise, osteoporosis becomes an increasingly important healthcare concern due its economic impact and harmful effects on human health, especially among women [1]. Adult bone structure is largely determined during the two first decades of life; thus factors that stimulate bone formation during childhood and adolescence have a major role in the prevention of osteoporosis later in life [2]. Particularly, sex, ethnicity, hormones, alcohol consumption, tobacco, nutrition, and exercise are among the most significant contributing factors that can influence bone acquisition during early life [3].

During the period of growth, physical activity (mostly weight-bearing activities) is a particularly relevant factor for achieving an optimum peak bone mass level, due to the positive osteogenic response [4, 5]. In a cross-sectional study, Ginty et al. [6] demonstrated that high-impact loading activities (such as jogging, playing tennis, football, rugby, basketball, and exercising with weights) for 1 h or more a day was associated with greater size-adjusted whole body (+3.4%) and total hip (+8.5%) bone mineral content (BMC) among male adolescents, compared to those with a median of 7 min∙day of participation in high-impact loading activities. Although weight-bearing physical activity during childhood and adolescence has been widely recognized to be beneficial for bone health [7], previous studies have focused predominantly on assessing the combined effect of different high-impact loading sports (mixing participants from different sports into the same bone-loading category) [8, 9]. Thus, the osteogenic effect of specific sports is less well understood.

Muscle and bone are inextricably linked not only mechanically but also genetically and molecularly. Recent studies demonstrated a molecular “cross talk” between muscle and bone, as both tissues release endocrine, paracrine, and autocrine factors that may mediate intercellular communication [10]. Although non-impact loading sports, such as swimming, are widely recognized to have no substantial positive effect on bone health [11, 12], they stimulate muscle contraction in a variety of muscles that can induce hypertrophy and also may potentially stimulate the molecular “cross talk” between muscle and bone [13]. Thus, exploring the effect of sports with clearly different bone-loading mechanisms, such as volleyball and swimming, may help to better understand the interaction between skeletal muscle and bone during growth.

Sex differences in bone quality and strength are well described [14]. By late puberty, boys have higher bone strength than girls, which is due mainly to the larger bone size in boys than in girls [15]. During aging, men have a greater periosteal apposition that increases bone size and offsets bone loss more than in women, yielding fewer males than females at risk for fracture in old age [16]. Thus, the relationship between physical activity and bone health is particularly relevant among females compared to males because they are at increased risk for osteoporosis and fracture later in life. At present the most effective sport modality for bone health promotion in girls is unknown, largely because of the confounding effects of biological maturation on bone development during adolescence [17, 18].

The aim of the current study was to compare total and regional bone (mineral content and density) and soft-tissue composition (fat and lean mass) between female adolescent swimmers and volleyball players. As the positive impact of sports participation on bone mass can be tempered by nutritional factors (such as calcium, protein and total caloric intake), differences in dietary intake were controlled for. It was hypothesized that volleyball players would have higher whole-body and regional BMC and bone mineral density (BMD) than swimmers; no differences in fat mass and lean mass were expected between sports.

## Methods

### Participants and procedures

The sample was composed of 46 female athletes (swimmers: *n*=20; volleyball players: *n*=26; Additional file 1) aged 14.5-17.4 years who were recruited voluntarily from seven competitive clubs in the Portuguese Midlands. The following inclusion criteria were considered: (*i*) chronological age less than 17.5 years; (*ii*) reaching menarche >1 year before testing; (*iii*) a minimum of 2 years of competitive participation at the national level in the sport; (*iv*) absence of medication usage that could affect bone metabolism; (*v*) absence of bone fractures.

All procedures were approved by the Ethics Committee of the Faculty of Sport Sciences and Physical Education of the University of Coimbra (CE/FCDEF-UC/00102014). The study was conducted in accordance with the Declaration of Helsinki for human studies of the World Medical Association. Participants were informed of the objectives and methodology and also that participation was voluntary and that they could withdraw from the experiment at any time. Parents or legal guardians and each participant signed an informed consent document. All measurements were completed in the same laboratory. The primary outcomes were whole-body and regional BMC, BMD, fat mass, and lean mass, measured by dual-energy X-ray absorptiometry (DXA) scans. Secondary outcomes included: (*i*) chronological age, calculated to the nearest 0.1 year by subtracting birth date from date of assessment; (*ii*) age at menarche, retrospectively self-reported; (*iii*) athlete’s training experience, obtained from coaches; (*iv*) dietary intake, obtained using food frequency questionnaires; (*v*) and, a brief anthropometric battery. For descriptive purposes, characteristics of the total sample are presented in Additional file 2 and Additional file 3.

### Training history

Information about formal years of participation and annual training sessions were obtained from coaches who maintained individual registration records. Swimmers participated in 4-6 training sessions per week (60-120 min∙session^-1^) and 1-2 competitions per month. Volleyball players participated in 3-4 training sessions per week, usually 90 min∙session^-1^ and 1 game∙week, usually Saturdays or Sundays. Competition calendars were usually October-May for swimming and September-July for volleyball.

### Dietary intake

Dietary intake was obtained using a structured semi-quantitative food frequency questionnaire [19] over the previous 12 months, comprised of 86 food items or beverage categories. This is a validated dietary instrument used frequently in Portugal and is based on the frequency of consumption of the main sources of proteins (%Kcal), carbohydrates (%Kcal), total fat (%Kcal), saturated fat (%Kcal), monounsaturated fat (%Kcal), polyunsaturated fatty (%Kcal), cholesterol (mg), fiber (g), ethanol (g), and calcium (mg). Variables taken into consideration in the study (i.e., protein, cholesterol and calcium) are in accordance with previous literature [17, 20, 21].

### Body size and skinfolds

The same experienced technician performed anthropometry according to recommended and standardized procedures [22]. Stature (Harpenden stadiometer, model 98.603, Holtain, Crosswell, UK) and sitting height (Harpenden sitting height table, model 98.607, Holtain, Crosswell, UK) were measured to the nearest 0.1 cm (leg length was calculated as the difference between the two). Body mass was measured to the nearest 0.1 kg using a SECA balance (model 770, Hanover, MD, USA). Stature and body mass were expressed as sex-age-specific z-scores for a reference population [23]. Individual z-scores were calculated based on the LMS parameters (Lambda for the skew, Mu for the median, and Sigma for the generalized coefficient of variation) constructed for the Centers for Disease Control and Prevention 2000 growth charts [24]. Corresponding percentiles were obtained from standard normal distribution tables. Seven skinfolds (tricipital, bicipital, subscapular, suprailiac, abdominal, anterior thigh and medial calf) were measured to the nearest 1 mm using a Lange caliper (Beta Technology, Ann Arbor, MI, USA). Technical errors of measurement for stature (0.29 cm), sitting height (0.30 cm), weight (0.19 kg), and skinfolds (0.74-1.04 mm) were well within the range of several health surveys in the United States and a variety of field surveys [25].

### Dual-energy x-ray absorptiometry

Absorptiometry (fan-beam Lunar DPX-PRO) was used to measure total body BMC (g), BMD (g∙cm^-2^), fat mass, and lean mass using standard or thick mode depending on body stature. Participants were placed in the supine position on the scanning table with the body aligned along the central horizontal axis. Arms were positioned parallel to, but not touching the body. Forearms were pronated with hands flat on the bed. Legs were fully extended, and feet were secured with a canvas and Velcro support to avoid foot movement during the scan acquisition. One skilled technician performed and analyzed all scans following the manufacturer’s guidelines (V 13.6 software) for patient positioning. Identical scanning parameters were used for each scan, and the output report considered bone area, BMC, BMD, lean soft tissue, and fat tissue. The regions of interest were manually positioned according to International Society for Clinical Densitometry guidelines and were apportioned as subhead (clavicle as reference), trunk, upper limbs and lower limbs. Scan analysis was performed using the Lunar Encore software (Version 13.6). The machine’s calibration was checked and passed on a daily basis using the Lunar calibration epoxy resin phantom.

### Data analysis

Descriptive statistics (mean, standard deviation, and range) were calculated for the total sample. Kolmogorov-Smirnov test was used to check variable distributions. When the assumptions of normality were violated, log-transformations were performed to reduce nonuniformity of error. Student t-tests for independent samples were used to compare athlete’s physical characteristics by sport. Cohen’s *d* effect sizes and thresholds (0.2, 0.6, 1.2, 2.0, 4.0 for trivial, small, moderate, large, very large and extremely large) were used to evaluate the magnitude of differences [26]. Pearson’s product moment correlation coefficients (*r*_*y,x*_) were calculated to examine the magnitude and direction of relationships among variables extracted from DXA (Y_i_) with age, training experience, and body size descriptors (X_i_), by type of sport. The magnitude of correlations was interpreted as follows [26]: trivial (*r*<0.1), small (0.1<*r*<0.3), moderate (0.3<*r*<0.5), large (0.5<*r*<0.7), very large (0.7<*r*<0.9), and nearly perfect (*r*>0.9). Multivariate analysis of covariance (MANCOVA) was used to determine significant differences between groups in total and regional bone and soft-tissue composition (fat and lean mass), after adjustments for age, training experience, and body size. The effect sizes for correlations (ES-*r*) were estimated using the square root of the ratio of the F-ratio squared and the difference between the F-value squared and degrees of freedom [27]. Coefficients were interpreted as follows: trivial (*r*<0.1), small (0.1<*r*<0.3), moderate (0.3<*r*<0.5), large (0.5<*r*<0.7), very large (0.7<*r*<0.9), and nearly perfect (*r*>0.9) [26]. Statistical significance was set to a *p-*value < 0.05. Statistical analyses were performed using the software IBM SPSS v.23 for Mac OS (SPSS Inc., IBM Company, NY, USA) and *GraphPad* Prism software (GraphPad Software, Inc., La Jolla, CA, USA).

## Results

Current age, age at menarche, training history, anthropometry, and dietary intake of swimmers and volleyball players are presented in Table 1. Swimmers and volleyball players did not differ significantly in chronological age but differed moderately in age at menarche [d=0.65 (t=2.121, *p*<0.05)]; volleyball players experienced the first menstruation 0.72 years earlier than swimmers. Swimmers had significantly more years of training [d=1.69 (t=-3.836, *p*<0.01)] and annual number of training sessions (i.e., training volume) [d=6.30 (t=20.814, *p*<0.01)] compared to volleyball players. Athletes had mean statures and mean body masses that approximate, respectively, the 53th and 68th age-specific percentiles for U.S. girls [23]. Mean BMI-for-age exceeded the ≥50th percentile in both groups (swimmers: 60th percentile; volleyball players: 73rd percentiles) [23]. Volleyball players were heavier (body mass: d=-0.79 (t=-2.596, *p*<0.01), with more subcutaneous adipose tissue than swimmers (suprailiac, abdominal and anterior thigh skinfolds: d=-0.67 to -1.06 (t=-2.233 to -3.556, p=0.031 to *p*<0.01). Volleyball players also had higher levels of cholesterol intake [d=0.87 (t=-2.485, *p*<0.018)] than swimmers.

**Table 1:** Means and standard deviations by type of sport (swimmers vs. volleyball players) on age, training experience, dietary intake, body size and skinfolds

	X: Sport		Comparisons^b^				
Dependent variables Y_i_	Swimming (*n*=20)	Volleyball (*n=*26)	mean difference (95%CI)	t-student		Magnitude effects	
				t-value	p	d	(description)
Y_1_: Chronological age (years)	15.71±0.93	16.20±0.77	-0.49 (-1.00; 0.02)	-1.957	0.057	-0.59	(small)
Y_2_: Age at menarche (years)	13.31±1.33	12.59±0.95	0.72 (0.04; 1.39)	2.121	0.040	0.65	(moderate)
Y_3_: Years of training (years)	8.9±3.9	4.1±1.8	4.8 (2.9; 6.7)	-3.836	<0.01	1.69	(large)
Y_4_: Annual number of training sessions (#)	298±34	115±26	182 (164; 200)	20.814	<0.01	6.30	(extremely large)
Y_5_: Energy intake ^a^ (Kcal/day)	2557±1188	3036±1188	-480 (-1305; 346)	-1.183	0.245	-0.42	(small)
Y_6_: Proteins ^a^ (%Kcal)	18.1±2.8	20.5±5.2	-2.4 (-5.5; 0.7)	-1.590	0.128	-0.62	(moderate)
Y_7_: Cholesterol ^a^ (mg)	353±174	527±241	-174 (-317; -32)	-2.485	0.018	-0.87	(moderate)
Y_8_: Calcium ^a^ (mg)	1141±556	1334±455	-192 (-550; 166)	-1.091	0.283	-0.39	(small)
Y_9_: Stature (cm)	161.3±4.4	164.2±6.0	-2.9 (-6.1; 0.3)	-1.821	0.075	-0.55	(small)
Y_10_: Body mass (kg)	55.0±5.6	61.0±9.0	-6.0 (-10.6; -1.3)	-2.596	0.013	-0.79	(moderate)
Y_11_: Skinfold triceps (mm)	17.9±6.0	20.4±3.9	-2.5 (-5.7; 0.6)	-1.642	0.111	-0.52	(small)
Y_12_: Skinfold subscapular (mm)	12.4±4.2	13.3±3.5	-1.0 (-3.3; 1.3)	-0.868	0.390	-0.24	(small)
Y_13_: Skinfold suprailiac (mm)	17.8±5.5	22.7±6.9	-4.9 (-8.7; -1.1)	-2.581	0.013	-0.79	(moderate)
Y_14_: Skinfold abdominal (mm)	17.6±6.1	22.0±7.1	-4.5 (-8.5; -0.4)	-2.233	0.031	-0.67	(moderate)
Y_15_: Skinfold thigh anterior (mm)	18.7±6.8	25.1±5.6	-6.5 (-10.1; -2.8)	-3.556	0.001	-1.06	(moderate)
Y_16_: Skinfold calf medial (mm)	17.9±5.3	17.7±4.8	0.20 (-2.8; 3.2)	0.113	0.910	0.04	(trivial)

*95%CI* 95% confidence intervals

^a^20 swimmers and 15 volleyball players completed the food questionnaire

^b^Results of comparisons between groups on chronological age, age at menarche, training experience, outputs obtained from the food questionnaire and anthropometry, mean differences, results of t-student test for independent samples and magnitude effect size (Cohen’s *d*)

**Fig. 1:**
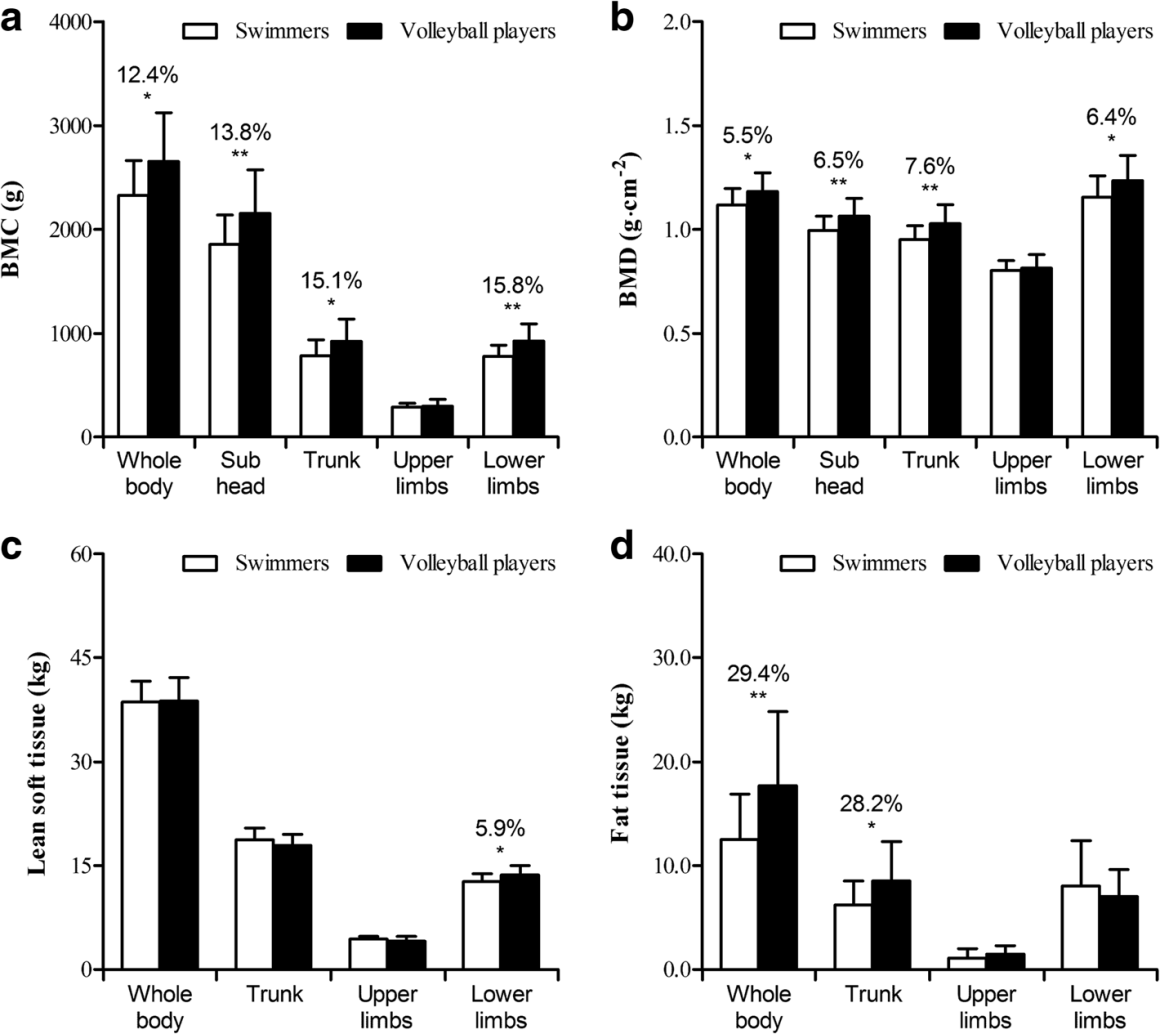
Bone mineral content (BMC, panel **a**), bone mineral density (BMD, panel **b**), lean soft tissue (panel **c**) and fat tissue (panel **d**) in female swimmers (white bars) and volleyball players (black bars). * indicates difference between the groups (*p*<0.05), ** *p*<0.01

Table 2 and Fig. 1 comprises the descriptive statistics for DXA whole body and regional body composition of swimmers and volleyball players. Overall, swimmers (BMC: 2328±338 g) and volleyball players (BMC: 2656±470 g) exceeded in 2.1 and 2.8 standard deviation scores, respectively, the average of international standards for whole body BMC of healthy adolescents of the same race, gender, stature and body mass [28]. Volleyball players had significantly greater BMC and BMD in the whole body [+12.4%: d=-0.80 (t=-2.637, *p*<0.05) and +5.5%: d=-0.80 (t=-2.574, *p*<0.05), respectively], subhead [+13.8%: d=-0.83 (t=-2.724, *p*<0.01) and +6.5%: d=-0.89 (t=-2.893, *p*<0.01), respectively], trunk [+15.1%: d=-0.76 (t=-2.491, *p*<0.05) and +7.6%: d=-0.98 (t=-3.233, *p*<0.01, respectively] and lower limbs [15.8%: d=-1.06 (t=-3.476, p<0.01) and +6.4%: d=-0.71 (t=-2.344, *p*<0.05), respectively] than swimmers. In addition, volleyball players had significantly greater lean soft tissue in the lower limbs [+5.9%: d=-0.72 (t=-2.217, *p*<0.05)], whole body [+29.4%: d=-0.87 (t=-2.853, *p*<0.01)], and trunk fat tissue [+28.2%: d=-0.73 (t=-2.456, *p*<0.05)].

**Table 2:** Means and standard deviations by type of sport (swimmers vs. volleyball players) on variables extracted from dual-energy x-ray absorptiometry

	X: Sport		Comparisons ^a^				
Dependent variables Y_i_	Swimming (*n=2*0)	Volleyball (*n=*26)	mean difference (95%CI)	t-student		Magnitude effects	
				t-value	p	d	(description)
Bone mineral content (g)							
Y_1_: Whole body	2328±338	2656±470	-328 (-578; -77)	-2.637	0.012	-0.80	(moderate)
Y_2_: Subhead	1856±284	2154±420	-298 (-518; -77)	-2.724	0.009	-0.83	(moderate)
Y_3_: Trunk	786±153	926±213	-140 (-254; -27)	-2.491	0.017	-0.76	(moderate)
Y_4_: Upper limbs	290±36	300±64	-10 (-42; 22)	-0.626	0.534	-0.19	(trivial)
Y_5_: Lower limbs	781±106	928±164	-147 (-232; -62)	-3.476	0.001	-1.06	(moderate)
Bone mineral density (g∙cm^-2^)							
Y_6_: Whole body	1.118±0.079	1.184±0.089	-0.065 (-0.116; -0.014)	-2.574	0.013	-0.80	(moderate)
Y_7_: Subhead	0.995±0.070	1.064±0.086	-0.069 (-0.116; -0.021)	-2.893	0.006	-0.89	(moderate)
Y_8_: Trunk	0.950±0.067	1.028±0.091	-0.078 (-0.127; -0.030)	-3.233	0.002	-0.98	(moderate)
Y_9_: Upper limbs	0.801±0.049	0.812±0.066	-0.011 (-0.046; 0.024)	-0.616	0.541	-0.19	(trivial)
Y_10_: Lower limbs	1.155±0.103	1.235±0.123	-0.080 (-0.149; -0.011)	-2.344	0.024	-0.71	(moderate)
Lean soft tissue (kg)							
Y_11_: Whole body	38.6±3.0	38.7±3.4	-0.1 (-2.0; 1.8)	-0.109	0.914	-0.03	(trivial)
Y_12_: Trunk	18.7±1.7	17.9±1.6	0.8 (-0.2; 1.8)	1.596	0.118	0.59	(small)
Y_13_: Upper limbs	4.4±0.4	4.1±0.7	0.3 (0.1; 0.6)	2.096	0.043	0.52	(small)
Y_14_: Lower limbs	12.7±1.1	13.6±1.4	-0.8 (-1.6; -0.1)	-2.217	0.032	-0.72	(moderate)
Fat tissue (kg)							
Y_15_: Whole body	12.5±4.4	17.7±7.1	-5.2 (-8.8; -1.5)	-2.853	0.007	-0.87	(moderate)
Y_16_: Trunk	6.2±2.3	8.5±3.8	-2.4 (-4.3; -0.4)	-2.456	0.018	-0.73	(moderate)
Y_17_: Upper limbs	1.1±0.9	1.5±0.8	-0.4 (-0.9; 0.1)	-1.602	0.116	-0.48	(small)
Y_18_: Lower limbs	8.0±4.4	7.0±2.6	0.9 (-1.3; 3.2)	0.865	0.394	0.29	(small)

*95%CI* 95% confidence intervals

^a^Results of comparisons between groups on variables extracted from the dual energy x-ray absorptiometry, mean differences, results of t-student test for independent samples and magnitude effect size (Cohen’s *d*)

Table 3 summarizes the interrelationship between age, training experience, and body size descriptors with variables extracted from DXA, by type of sport. In swimmers, BMC, BMD (whole body, subhead, trunk, and upper limbs) and fat tissue (whole body, trunk, and lower limbs) are moderately to largely correlated with CA (*r*=+0.46 to +0.59, *p*<0.05). No significant associations were noted within the same variables for volleyball players. With one exception – lower limbs lean soft tissue in volleyball players (*r*=+0.40, *p*<0.05) – no correlations were found between age at menarche and total and regional bone mineral and tissue composition. Training experience in swimmers is positively related to the upper limbs BMC (*r*=+0.49, p<0.05) and fat tissue (*r*=+0.49, *p*<0.05), and lower limbs fat tissue (*r*=+0.52, *p*<0.05). In volleyball players, years of training only correlated significantly with lower limbs BMD (*r*=+0.43, *p*<0.05). Correlations between stature and BMC in swimmers are large to very large (*r*=+0.64 to +0.82, *p*<0.01) and are generally higher than those for BMD (*r*=+0.57 to +0.66, *p*<0.01) and lean soft tissue (whole body: *r*=+0.52, *p*<0.05; lower limbs: *r*=+0.70, *p*<0.01). The BMC [with one exception: lower limbs (*r*=+0.46, *p*<0.05)] and BMD of volleyball players are not significantly related to stature. In contrast to bone mineral parameters, stature is largely related to lean soft tissue (whole body: *r*=+0.63, *p*<0.01; trunk: *r*=+0.64, *p*<0.01; lower limbs: *r*=+0.61, *p*<0.01). Although the correlation data between body mass and variables extracted from DXA for swimmers and volleyball players are somewhat different, they reflect moderate to very large [BMC, BMD, lean soft tissue (whole body, upper and lower limbs) and fat tissue (whole body and trunk)] and moderate to nearly perfect [BMC, BMD (whole body, subhead and trunk), lean soft tissue (whole body, upper and lower limbs), and fat tissue] positive associations (*p*<0.05), respectively.

**Table 3:** Correlations between age, training experience, and body size descriptors (Xi) with variables extracted from dual-energy x-ray absorptiometry (Yi) by type of sport (swimmers and volleyball players)

	Correlation (Xi, Yi)									
	X1: Chronological age		X2: Age at menarche		X3: Years of training		X4: Stature		X5: Body mass	
	Swimming (*n*=20)	Volleyball (*n*=26)	Swimming (*n*=20)	Volleyball (*n*=26)	Swimming (*n*=20)	Volleyball (*n*=26)	Swimming (*n*=20)	Volleyball (*n*=26)	Swimming (*n*=20)	Volleyball (*n*=26)
	*r* (95%CI)	*r* (95%CI)	*r* (95%CI)	*r* (95%CI)	*r* (95%CI)	*r* (95%CI)	*r* (95%CI)	*r* (95%CI)	*r* (95%CI)	*r* (95%CI)
Bone mineral content (g)										
Y_1_: Whole body	0.51* (0.11; 0.76)	0.20 (-0.14; 0.55)	0.11 (-0.31; 0.46)	0.18 (-0.25; 0.49)	0.31 (-0.05; 0.64)	0.19 (-0.23; 0.65)	0.81** (0.53; 0.92)	0.32 (-0.21; 0.63)	0.85** (0.69; 0.94)	0.71** (0.32; 0.87)
Y_2_: Subhead	0.53* (0.14; 0.78)	0.16 (-0.14; 0.52)	0.14 (-0.30; 0.49)	0.19 (-0.23; 0.50)	0.31 (-0.05; 0.65)	0.18 (-0.24; 0.65)	0.82** (0.57; 0.93)	0.35 (-0.17; 0.64)	0.87** (0.71; 0.95)	0.73** (0.33; 0.89)
Y_3_: Trunk	0.48* (0.06; 0.75)	0.11 (-0.24; 0.49)	0.14 (-0.33; 0.48)	0.12 (-0.24; 0.49)	0.26 (-0.10; 0.61)	0.14 (-0.26; 0.60)	0.82** (0.56; 0.92)	0.26 (-0.35; 0.61)	0.86** (0.71; 0.93)	0.76** (0.34; 0.88)
Y_4_: Upper limbs	0.59** (0.25; 0.82)	0.14 (-0.13; 0.46)	0.15 (-0.36; 0.56)	0.11 (-0.39; 0.52)	0.49* (0.08; 0.74)	0.28 (-0.12; 0.67)	0.64** (0.38; 0.81)	0.25 (-0.25; 0.58)	0.76** (0.57; 0.90)	0.48* (0.01; 0.75)
Y_5_: Lower limbs	0.52* (0.15; 0.78)	0.22 (-0.05; 0.53)	0.11 (-0.28; 0.49)	0.27 (-0.14; 0.57)	0.29 (-0.09; 0.62)	0.17 (-0.25; 0.65)	0.80** (0.52; 0.92)	0.46* (0.07; 0.70)	0.82** (0.64; 0.93)	0.68** (0.29; 0.86)
Bone mineral density (g∙cm^-2^)										
Y_6_: Whole body	0.46* (0.06; 0.75)	0.21 (-0.24; 0.62)	-0.03 (-0.35; 0.31)	0.19 (-0.29; 0.53)	0.33 (-0.11; 0.69)	0.36 (-0.05; 0.72)	0.63** (0.22; 0.83)	0.01 (-0.47; 0.41)	0.67** (0.36; 0.86)	0.41* (0.01; 0.69)
Y_7_: Subhead	0.50* (0.11; 0.80)	0.13 (-0.28; 0.56)	0.04 (-0.29; 0.39)	0.21 (-0.26; 0.56)	0.31 (-0.17; 0.69)	0.37 (-0.04; 0.75)	0.66** (0.30; 0.86)	0.10 (-0.41; 0.47)	0.70** (0.43; 0.87)	0.49* (0.06; 0.75)
Y_8_: Trunk	0.52* (0.09; 0.80)	0.08 (-0.30; 0.50)	0.08 (-0.30; 0.39)	0.09 (-0.40; 0.46)	0.28 (-0.20; 0.68)	0.36 (-0.06; 0.74)	0.65** (0.26; 0.85)	0.04 (-0.50; 0.46)	0.69** (0.42; 0.85)	0.57** (0.10; 0.77)
Y_9_: Upper limbs	0.51* (0.17; 0.73)	0.08 (-0.42; 0.54)	-0.07 (-0.44; 0.30)	0.33 (-0.28; 0.71)	0.45* (0.05; 0.71)	0.21 (-0.11; 0.56)	0.57** (0.26; 0.79)	-0.23 (-0.63; 0.13)	0.67** (0.39; 0.91)	0.24 (-0.16; 0.58)
Y_10_: Lower limbs	0.40 (0.01; 0.81)	0.13 (-0.27; 0.53)	0.01 (-0.35; 0.44)	0.20 (-0.21; 0.54)	0.27 (-0.21; 0.68)	0.43* (-0.03; 0.77)	0.59** (0.28; 0.81)	0.19 (-0.34; 0.55)	0.63** (0.34; 0.83)	0.37 (-0.13; 0.72)
Lean soft tissue (kg)										
Y_11_: Whole body	0.18 (-0.32; 0.54)	0.30 (-0.02; 0.61)	0.08 (-0.58; 0.56)	0.38 (0.07; 0.65)	0.02 (-0.38; 0.38)	0.38 (0.02; 0.66)	0.52* (0.05; 0.79)	0.63** (0.40; 0.88)	0.46* (0.07; 0.72)	0.42* (0.01; 0.67)
Y_12_: Trunk	-0.05 (-0.57; 0.41)	0.29 (-0.13; 0.62)	0.08 (-0.53; 0.54)	0.34 (0.04; 0.69)	-0.08 (-0.45; 0.36)	0.37 (0.09; 0.62)	0.21 (-0.46; 0.71)	0.64** (0.44; 0.80)	0.20 (-0.41; 0.63)	0.32 (-0.11; 0.60)
Y_13_: Upper limbs	0.41 (-0.04; 0.69)	0.14 (-0.15; 0.45)	0.12 (-0.48; 0.54)	0.13 (-0.27; 0.50)	0.33 (-0.08; 0.65)	0.30 (-0.06; 0.62)	0.31 (0.01; 0.62)	0.17 (-0.23; 0.57)	0.51* (0.17; 0.74)	0.41* (-0.06; 0.70)
Y_14_: Lower limbs	0.19 (-0.34; 0.61)	0.30 (0.02; 0.57)	0.26 (-0.20; 0.67)	0.40* (-0.02; 0.70)	-0.08 (0.47; 0.29)	0.24 (-0.16; 0.58)	0.70** (0.36; 0.88)	0.61** (0.35; 0.79)	0.56** (0.22; 0.77)	0.41* (0.03; 0.67)
Fat tissue (kg)										
Y_15_: Whole body	0.48* (0.01; 0.75)	0.04 (-0.24; 0.37)	0.06 (-0.45; 0.53)	-0.02 (-0.27; 0.22)	0.41 (0.09; 0.70)	-0.06 (-0.57; 0.38)	0.41 (-0.15; 0.78)	0.14 (-0.38; 0.51)	0.84** (0.61; 0.95)	0.94** (0.77; 0.97)
Y_16_: Trunk	0.48* (0.03; 0.74)	0.08 (-0.21; 0.39)	0.04 (-0.47; 0.51)	-0.02 (-0.24; 0.21)	0.44 (0.15; 0.72)	-0.07 (-0.55; 0.35)	0.43 (-0.12; 0.78)	0.15 (-0.39; 0.52)	0.85** (0.63; 0.95)	0.93** (0.74; 0.97)
Y_17_: Upper limbs	0.38 (0.01; 0.81)	-0.05 (-0.36; 0.27)	0.20 (-0.24; 0.53)	-0.04 (-0.35; 0.20)	0.49* (0.35; 0.72)	-0.01 (-0.59; 0.42)	-0.27 (-0.66; 0.63)	0.17 (-0.30; 0.50)	0.05 (-0.46; 0.84)	0.86** (0.60; 0.94)
Y_18_: Lower limbs	0.46* (0.05; 0.74)	0.02 (-0.26; 0.34)	0.16 (-0.33; 0.63)	-0.01 (-0.32; 0.27)	0.52* (0.15; 0.83)	-0.03 (-0.55; 0.38)	0.23 (-0.26; 0.64)	0.11 (-0.36; 0.47)	0.53 (0.03; 0.82)	0.88** (0.67; 0.95)

*r* correlation coefficients, *95%CI* 95% confidence intervals

*p < 0.05, **p < 0.01

Sport-related variation for total and regional bone and soft-tissue composition, when chronological age, age at menarche, training experience, stature and body mass were statistically controlled by MANCOVA is presented in Table 4. Moderate differences (ES-*r*=0.32) between swimmers and volleyball players persisted for BMD at the lower limbs (0.098 g∙cm^-2^, 7.8%).

**Table 4:** Estimated marginal means controlling for age, training experience, and body size descriptors to examine variation associated to type of sport in variables extracted from dual-energy x-ray absorptiometry

	X: Sport					
Dependent variables Yi	Swimming^a^ (*n*=20)	Volleyball^a^ (*n*=26)	MANCOVA^b^		Magnitude effect	
			F	*p*	ES-*r*	(descriptive)
Bone mineral content (g)						
Y_1_: Whole body	2476±84	2615±69	1.113	0.298	0.167	(small)
Y_2_: Subhead	1982±71	2118±58	1.496	0.229	0.192	(small)
Y_3_: Trunk	858±37	897±30	0.461	0.501	0.110	(small)
Y_4_: Upper limbs	296±14	304±11	0.153	0.698	0.063	(small)
Y_5_: Lower limbs	830±28	916±23	3.822	0.058	0.298	(small)
Bone mineral density (g∙cm^-2^)						
Y_6_: Whole body	1.132±0.024	1.190±0.019	2.414	0.128	0.241	(small)
Y_7_: Subhead	1.005±0.021	1.069±0.018	3.648	0.063	0.293	(small)
Y_8_: Trunk	0.965±0.021	1.020±0.018	2.617	0.114	0.251	(small)
Y_9_: Upper limbs	0.800±0.017	0.821±0.014	0.656	0.423	0.130	(small)
Y_10_: Lower limbs	1.160±0.030	1.258±0.025	4.306	0.045	0.315	(moderate)
Lean soft tissue (kg)						
Y_11_: Whole body	39.0±0.8	39.1±0.6	0.006	0.937	0.032	(trivial)
Y_12_: Trunk	18.7±0.5	18.2±0.4	0.518	0.476	0.114	(small)
Y_13_: Upper limbs	4.4±0.2	4.1±0.1	1.537	0.223	0.195	(small)
Y_14_: Lower limbs	13.0±0.3	13.7±0.2	2.769	0.104	0.257	(small)
Fat tissue (kg)						
Y_15_: Whole body	15.9±0.7	15.7±0.6	0.017	0.896	0.032	(trivial)
Y_16_: Trunk	8.1±0.4	7.6±0.3	0.616	0.437	0.126	(small)
Y_17_: Upper limbs	1.1±0.2	1.5±0.2	1.620	0.211	0.200	(small)
Y_18_: Lower limbs	8.4±0.9	6.9±0.7	1.334	0.255	0.182	(small)

*ES-r* effect size correlation

^a^Data presented as estimated marginal means ± standard error

^b^MANCOVA models adjusted by chronological age, age at menarche, years of training, stature, and body mass

## Discussion

This study showed that volleyball players presented greater BMC and BMD, in the whole body, and greater lean soft tissue in the lower limbs with respect to swimmers. Although swimming stimulates muscle hypertrophy [29], our results showed that swimmers had moderately less skinfold thickness (a measure of subcutaneous fat), whole body and trunk fat tissue. Differences between groups in lean mass were mostly small. Thus, the mechanical and non-mechanical stimuli associated with swimming may not be sufficient to trigger the responsiveness of bone cells. After adjustments for potential confounders (i.e., chronological age, age at menarche, years of training, stature, and body mass) the bone content, lean soft tissue and fat tissue were similar between groups. Differences persisted for the lower limbs, with volleyball players presenting higher BMD compared to swimmers.

A secular decline in the age at menarche occurred in the Portuguese population [30]. Findings suggest that the decline is associated, to a large extent, with a reduction in the number of girls who mature late [25]. Allowing for normal variability, training is not related to age at menarche in athletes [31] and variation in mean ages at menarche within a sport is especially evident in swimmers and volleyball players [25]. Mean ages at menarche were 13.31±1.33 years and 12.59±0.95 years for swimmers and volleyball players, respectively. Volleyball players approximates the mean age of menarche for Portuguese school girls calculated using recall methods (i.e., 12.53±1.27 years) [30]. Although there is a difference of 0.72 years between groups’ means, about two-thirds of the present sample attains menarche between 12.0 and 14.0 years. The limited variability may explain the reduced interrelationships between age at menarche with variables extracted from DXA (see Table 3), suggesting that they are somewhat independent.

The current sample has a mean stature and mean body mass which approximate, respectively, the 53th and 68th age-specific percentiles for U.S. girls [23]. The trend for elevated mass-for-stature likely reflects the advanced maturity status of the athletes [31]. Mean BMI-for-age exceeded the ≥50th percentile in both groups (swimmers: 60th percentile; volleyball players: 73rd percentiles) [23]. This was consistent with observations for other samples of youth swimmers and volleyball players [25, 32]. In the current study, dietary data suggested differences only for cholesterol intake with higher amounts being consumed by volleyball players compared to swimmers. Volleyball players were significantly heavier (+6 kg) but not taller than swimmers. Differences between groups in the components assessed by whole-body DXA were moderate for mineral content (328 g) and fat tissue (5200 g) and trivial for lean soft tissue (100 g). Allowing for the limitation of the comparison, swimmers and volleyball players exceeded in 2.1 and 2.8 standard deviation scores, respectively, the average of international standards for whole body BMC of healthy adolescents [28]. This suggests greater BMC of female adolescent swimmers and volleyball players compared to healthy female adolescents, likely with positive effects on bone health later in life.

Reduced lean mass constitutes one of the most relevant determinants of risk for low BMD in female adolescent runners [33], while lean mass is related to BMD gains and bone geometry changes in female soccer players [18]. As previously described, part of the osteogenic effect attributed to sport participation may be related to the increase in muscle mass, and subsequent effect on bone cells [12, 34]. Correlations between primary and secondary outcomes in swimmers and volleyball players are summarized in Table 3. Results indicate the complexities involved in attempting to partition out the contribution of age, training and body size, which are often overlooked in comparisons of bone and soft tissue between athletes of different sports.

Bone is a component of body composition that is a focus of attention specifically in the context of preventing osteoporosis later in life [1, 2]. In general, the more mineral accumulated in the skeleton during growth and maturation, the better off the individual will be several decades later when mineral content of the skeleton begins to decline [2, 21]. Evidence from cross-sectional studies suggested that the peak of bone mass acquisition is reached during adolescent years and significantly affects the BMC observed in adulthood [2, 12, 34]. It has been noted that active adolescent males had 8-10% more adjusted BMC at the total body, total hip and femoral neck (*p*<0.05) in young adulthood and active adolescent females had 9-10% more adjusted BMC at the total hip and femoral neck [21]. In the present study, years of training were positively related to the upper limbs BMC (*r*=+0.49, *p*<0.05) in swimmers and with lower limbs BMD (*r*=+0.43, *p*<0.05) in volleyball players. All together, this would suggest that the adoption of routines of physical activity including exercise and sport participation during adolescence may itself mediate enhanced skeleton formation, and these benefits are maintained into young adulthood, which may help prevent musculoskeletal diseases, such as osteoporosis, during old age.

The lower values of BMD in swimmers when compared to other sports have been investigated over the last years [29]. Although the mechanisms are not entirely clear, recent evidence point to several possible explanations. It has been theorized that muscle forces produced during sports such as swimming and cycling may not exceed the minimum effective strain stimulus threshold to induce an osteogenic effect [35, 36]. The most relevant aspect of the non-significant effect of swimming on bone gains is due its movement in a “hypogravity” environment [12, 33] for large amount of time per week [18]. The extensive time spent in the water by swimmers may also limit the time they have available to perform other sports, including weight-bearing activities during the day.

The apparent benefits of high-impact loading sport (i.e., volleyball) on BMC and BMD in the present study seemed to be specific to the trunk and lower limbs while differences between volleyball players and swimmers for bone mineral parameters in the upper limbs were trivial. Mechanical loading leads to bone remodelling, adapting the bone structure in response to the mechanical demands [12]. The stress generated by physical exercise on bone stimulates the collagen alignment in the sites directly affected by the activity, leading to higher bone strength [34]. The high quantity of jumps required during a volleyball practice may explain, at least in part, the differences observed for BMD at the lower limbs compared to swimmers. Ferry et al. [18] noted the same effect on lower limbs among girls engaged in soccer practice, in which jumps, kicks, and sprints are commonly performed. In addition, bone cells become desensitized to prolonged mechanical stimulation [37], thus incorporating periods of rest between short vigorous skeletal loading sessions may be a valid strategy to promote osteogenesis. Volleyball practice is characterized by intermittent movement (acceleration and jumps), which may also explain the higher bone mass observed in the female volleyball players compared to swimmers.

A major strength of this study was the inclusion of under-researched late-adolescent female athletes (i.e., volleyball players and swimmers). A further strength of the study was the use of DXA which is considered the safest and most appropriate imaging modality to access body composition and bone status. However, the current investigation is also not without limitations. First, the cross-sectional design prevents comment on causation. The sample size is fairly small, and we were unable to control for participation in other sports, or factors known to affect bone mineral density. Second, no regional site scans at the lumbar spine and proximal femur were performed, and no accurate geometrical properties were captured by DXA. Further research might attempt to control for the influences of biological maturity when seeking to determine the effects of specific physical activities on bone health among young female athletes. This will help inform programs and strategies to enhance bone health during the adolescent period of growth and development, leading to prevention of osteoporosis in later years.

## Conclusions

In conclusion, volleyball players had greater BMD at the lower limbs when compared to swimmers. The results support the fact that 3 to 4 times per week of high impact loading activities are associated with higher bone mineral density at regions of interest. The observed skeletal benefits may also translate in positive changes in bone geometry and quality, this providing a substantial increase in bone strength. This observational study provides practical implications for inactive young individuals, young athletes of non-impact loading sports (including swimming) and coaches who can benefit from complementing training routines with osteogenic weight-bearing activities [29], such as resistance, strength and plyometric training.

## Additional files

Additional file 1: Dataset used in this Research. SPORT: sport group; H: stature; SH: sitting height; LL: leg length; BM: body mass; SKT: skinfold triceps; SKSB: skinfold subscapular; SKSP: skinfold suprailiac; SKAB: skinfold abdominal; SKTA: skinfolds thigh anterior; SKCM: skinfolds calf medial; WBBMC: whole body bone mineral content; SHBMC: subhead bone mineral content; TKBMC: trunk bone mineral content; ULBMC: upper limbs bone mineral content; LLBMC: lower limbs bone mineral content; WBBMD: whole body bone mineral density; SHBMD: subhead bone mineral density; TKBMD: trunk bone mineral density; ULBMD: upper limbs bone mineral density; LLBMD: lower limbs bone mineral density; WBLST: whole body lean soft tissue; TKLST: trunk lean soft tissue; ULLST: upper limbs lean soft tissue; LLLST: lower limbs lean soft tissue; WBFT: whole body fat tissue; TKFT: trunk fat tissue; ULFT: upper limbs fat tissue; LLFT: lower limbs fat tissue.

Additional file 2: Descriptive statistics for chronological age, age at menarche, years of training, annual sessions, outputs from the food questionnaire and anthropometry for the total sample (*n*=46).

Additional file 3: Descriptive statistics for outputs obtained from the DXA assessment for the whole body and regions of interest for the total sample (*n*=46).

## Abbreviations

MANCOVA: multivariate analysis of covariance
BMC: bone mineral content
BMD: bone mineral density
CI: confidence intervals
DXA: Dual-energy X-ray absorptiometry
ES-*r*: effect size correlations
K-S: Kolmogorov-Smirnov
SEM: standard error of the mean

## References

[1] Johnell O, Kanis JA (2006). An estimate of the worldwide prevalence and disability associated with osteoporotic fractures. Osteoporos Int.

[2] Rizzoli R, Bianchi ML, Garabedian M, McKay HA, Moreno LA (2010). Maximizing bone mineral mass gain during growth for the prevention of fractures in the adolescents and the elderly. Bone.

[3] Bonjour JP, Chevalley T, Rizzoli R, Ferrari S (2007). Gene-environment interactions in the skeletal response to nutrition and exercise during growth. Med Sport Sci.

[4] Behringer M, Gruetzner S, McCourt M, Mester J (2014). Effects of weight-bearing activities on bone mineral content and density in children and adolescents: a meta-analysis. J Bone Miner Res.

[5] Wallace IJ, Kwaczala AT, Judex S, Demes B, Carlson KJ (2013). Physical activity engendering loads from diverse directions augments the growing skeleton. J Musculoskelet Neuronal Interact.

[6] Ginty F, Rennie KL, Mills L, Stear S, Jones S, Prentice A (2005). Positive, site-specific associations between bone mineral status, fitness, and time spent at high-impact activities in 16- to 18-year-old boys. Bone.

[7] Boreham CA, McKay HA (2011). Physical activity in childhood and bone health. Br J Sports Med.

[8] Gruodyte R, Jurimae J, Cicchella A, Stefanelli C, Passariello C, Jurimae T (2010). Adipocytokines and bone mineral density in adolescent female athletes. Acta Paediatr.

[9] Nichols JF, Rauh MJ, Barrack MT, Barkai HS (2007). Bone mineral density in female high school athletes: interactions of menstrual function and type of mechanical loading. Bone.

[10] Pedersen BK, Febbraio MA (2012). Muscles, exercise and obesity: skeletal muscle as a secretory organ. Nat Rev Endocrinol.

[11] Scofield KL, Hecht S (2012). Bone health in endurance athletes: runners, cyclists, and swimmers. Current Sports Med Rep.

[12] Tenforde AS, Fredericson M (2011). Influence of sports participation on bone health in the young athlete: a review of the literature. PMR.

[13] Sartori R, Sandri M (2015). BMPs and the muscle-bone connection. Bone.

[14] Seeman E (2001). Clinical review 137: Sexual dimorphism in skeletal size, density, and strength. J Clin Endocrinol Metab.

[15] Kirmani S, Christen D, van Lenthe GH, Fischer PR, Bouxsein ML, McCready LK (2009). Bone structure at the distal radius during adolescent growth. J Bone Miner Res.

[16] Duan Y, Turner CH, Kim BT, Seeman E (2001). Sexual dimorphism in vertebral fragility is more the result of gender differences in age-related bone gain than bone loss. J Bone Miner Res.

[17] Burt LA, Naughton GA, Greene DA, Courteix D, Ducher G (2012). Non-elite gymnastics participation is associated with greater bone strength, muscle size, and function in pre- and early pubertal girls. Osteoporos Int.

[18] Ferry B, Lespessailles E, Rochcongar P, Duclos M, Courteix D (2013). Bone health during late adolescence: effects of an 8-month training program on bone geometry in female athletes. Joint Bone Spine.

[19] Lopes C, Aro A, Azevedo A, Ramos E, Barros H (2007). Intake and adipose tissue composition of fatty acids and risk of myocardial infarction in a male Portuguese community sample. J Am Diet Assoc.

[20] Agostinete RR, Duarte JP, Valente-dos-Santos J, Coelho-e-Silva MJ, Tavares OM, Conde JM (2017). Bone tissue, blood lipids and inflammatory profiles in adolescent male athletes from sports contrasting in mechanical load. PLoS One.

[21] Baxter-Jones AD, Kontulainen SA, Faulkner RA, Bailey DA (2008). A longitudinal study of the relationship of physical activity to bone mineral accrual from adolescence to young adulthood. Bone.

[22] Lohman T, Roche AF, Martorell R (1988). Anthropometric standardization reference manual.

[23] Kuczmarski RJ, Ogden CL, Guo SS, Grummer-Strawn LM, Flegal KM, Mei Z (2002). 2000 CDC growth charts for the United States: methods and development. Vital Health Stat.

[24] Flegal KM, Cole TJ (2013). Construction of LMS parameters for the Centers for Disease Control and Prevention 2000 growth charts. Natl Health Stat Rep.

[25] Malina RM, Bouchard C, Bar-Or O (2004). Growth, maturation, and physical activity.

[26] Hopkins WG, Marshall SW, Batterham AM, Hanin J (2009). Progressive statistics for studies in sports medicine and exercise science. Med Sci Sports Exerc.

[27] Rosnow RL, Rassithal R (1996). Computing contrast, effect sizes and conternulls on other people is published data: General procedures for research consumer. Psychol Methods.

[28] Baxter-Jones AD, Burrows M, Bachrach LK, Lloyd T, Petit M, Macdonald H (2010). International longitudinal pediatric reference standards for bone mineral content. Bone.

[29] Gomez-Bruton A, Gonzalez-Aguero A, Gomez-Cabello A, Matute-Llorente A, Casajus JA, Vicente-Rodriguez G (2015). The effects of swimming training on bone tissue in adolescence. Scand J Med Sci Sports.

[30] Padez C, Rocha MA (2003). Age at menarche in Coimbra (Portugal) school girls: a note on the secular changes. Ann Hum Biol.

[31] Malina RM, Rogol AD, Cumming SP, Coelho e Silva MJ, Figueiredo AJ (2015). Biological maturation of youth athletes: assessment and implications. Br J Sports Med.

[32] Santos DA, Dawson JA, Matias CN, Rocha PM, Minderico CS, Allison DB (2014). Reference values for body composition and anthropometric measurements in athletes. PLoS One.

[33] Tenforde AS, Fredericson M, Sayres LC, Cutti P, Sainani KL (2015). Identifying sex-specific risk factors for low bone mineral density in adolescent runners. Am J Sports Med.

[34] Kini U, Nandeesh BN, Fogelman I, Gnanasegaran G, van der Wall H (2012). Physiology of Bone Formation, Remodeling, and Metabolism. Radionuclide and Hybrid Bone Imaging.

[35] Fehling PC, Alekel L, Clasey J, Rector A, Stillman RJ (1995). A comparison of bone mineral densities among female athletes in impact loading and active loading sports. Bone.

[36] Heinonen A, Oja P, Kannus P, Sievanen H, Manttari A, Vuori I (1993). Bone mineral density of female athletes in different sports. Bone Miner.

[37] Umemura Y, Ishiko T, Yamauchi T, Kurono M, Mashiko S (1997). Five jumps per day increase bone mass and breaking force in rats. J Bone Miner Res.

