# Supplementary material for: Total and regional bone mineral and tissue composition in female adolescent athletes: comparison between volleyball players and swimmers
Source: BMC Pediatr. 2018 Jul 3;18:212. doi: 10.1186/s12887-018-1182-z (PMC6031185; doi:10.1186/s12887-018-1182-z)
Supplement: Supplementary file 2 — Descriptive statistics for chronological age, age at menarche, years of training, annual sessions, outputs from the food questionnaire and anthropometry for the total sample (n=46). [file 12887_2018_1182_MOESM2_ESM.docx]

**Additional file 2** Descriptive statistics for chronological age, age at menarche, years of training, annual sessions, outputs from the food questionnaire and anthropometry for the total sample (n=46)

|  | Descriptive | | | | | | | Normality  (Kolmogorov-Smirnov) | |
| --- | --- | --- | --- | --- | --- | --- | --- | --- | --- |
|  | Range | | Mean | | | | Standard deviation |  |  |
|  | minimum | maximum | value | SEM | 95% CL | |  |  |  |
|  |  |  |  |  | lower | upper |  | K-S value | p |
|  |  |  |  |  |  |  |  |  |  |
| Chronological age (years) | 14.50 | 17.48 | 15.99 | 0.13 | 15.73 | 16.24 | 0.87 | 0.108 | 0.200 |
| Age at menarche (years) | 10.74 | 16.52 | 12.90 | 0.17 | 12.55 | 13.25 | 1.18 | 0.082 | 0.200 |
| Years of training (years) | 2 | 11 | 6.2 | 0.6 | 5.1 | 7.3 | 3.7 | 0.169 | 0.002 |
| Annual number of training sessions (#) | 58 | 350 | 195 | 14 | 166 | 223 | 96 | 0.225 | 0.000 |
|  |  |  |  |  |  |  |  |  |  |
| Energy intake (Kcal/day) | 1145 | 5710 | 2762 | 202 | 2352 | 3173 | 1195 | 0.134 | 0.115 |
| Proteins (%Kcal) | 12.0 | 29.0 | 19.1 | 0.7 | 17.7 | 20.5 | 4.1 | 0.207 | 0.001 |
| Cholesterol (mg) | 169 | 1166 | 427 | 37 | 352 | 503 | 220 | 0.176 | 0.008 |
| Calcium (mg) | 435 | 2620 | 1224 | 87 | 1046 | 1401 | 517 | 0.122 | 0.200 |
|  |  |  |  |  |  |  |  |  |  |
| Stature (cm) | 151.7 | 172.5 | 163.0 | 0.8 | 161.3 | 164.6 | 5.5 | 0.094 | 0.200 |
| Sitting height (cm) | 71.3 | 95.1 | 86.8 | 0.6 | 85.5 | 88.1 | 4.4 | 0.128 | 0.055 |
| Leg length (cm) | 67.9 | 83.0 | 76.1 | 0.5 | 75.1 | 77.1 | 3.4 | 0.072 | 0.200 |
| Body mass (kg) | 42.0 | 88.1 | 58.4 | 1.2 | 56.0 | 60.8 | 8.2 | 0.106 | 0.200 |
| Skinfold triceps (mm) | 8 | 29 | 19.3 | 0.7 | 17.8 | 20.8 | 5.0 | 0.112 | 0.188 |
| Skinfold subscapular (mm) | 7 | 22 | 12.9 | 0.6 | 11.8 | 14.1 | 3.8 | 0.137 | 0.030 |
| Skinfold suprailiac (mm) | 10 | 35 | 20.6 | 1.0 | 18.6 | 22.6 | 6.8 | 0.120 | 0.096 |
| Skinfold abdominal (mm) | 7 | 39 | 20.1 | 1.0 | 18.0 | 22.1 | 7.0 | 0.109 | 0.200 |
| Skinfolds thigh anterior (mm) | 6 | 38 | 22.3 | 1.0 | 20.3 | 24.3 | 6.9 | 0.105 | 0.200 |
| Skinfolds calf medial (mm) | 9 | 26 | 17.8 | 0.7 | 16.3 | 19.3 | 5.0 | 0.135 | 0.035 |
|  |  |  |  |  |  |  |  |  |  |

*SEM* standard error of the mean, *95%CL* 95% confidence limits
