# Supplementary material for: Total and regional bone mineral and tissue composition in female adolescent athletes: comparison between volleyball players and swimmers
Source: BMC Pediatr. 2018 Jul 3;18:212. doi: 10.1186/s12887-018-1182-z (PMC6031185; doi:10.1186/s12887-018-1182-z)
Supplement: Supplementary file 3 — Descriptive statistics for outputs obtained from the DXA assessment for the whole body and regions of interest for the total sample (n=46). [file 12887_2018_1182_MOESM3_ESM.docx]

**Additional file 3** Descriptive statistics for outputs obtained from the DXA assessment for the whole body and regions of interest for the total sample (n=46)

|  | Descriptive | | | | | | | Normality  (Kolmogorov-Smirnov) | |
| --- | --- | --- | --- | --- | --- | --- | --- | --- | --- |
|  | Range | | Mean | | | | Standard deviation |  |  |
|  | minimum | maximum | value | SEM | 95% CL | |  |  |  |
|  |  |  |  |  | lower | upper |  | K-S value | p |
|  |  |  |  |  |  |  |  |  |  |
| Bone mineral content |  |  |  |  |  |  |  |  |  |
| Whole body (g) | 1748 | 4006 | 2513 | 66 | 2381 | 2645 | 445 | 0.087 | 0.200 |
| Subhead (g) | 1398 | 3403 | 2025 | 58 | 1908 | 2141 | 393 | 0.092 | 0.200 |
| Trunk (g) | 552 | 1554 | 865 | 29 | 806 | 925 | 200 | 0.099 | 0.200 |
| Upper limbs (g) | 164 | 441 | 295 | 8 | 280 | 311 | 53 | 0.129 | 0.052 |
| Lower limbs (g) | 568 | 1413 | 864 | 23 | 817 | 911 | 158 | 0.079 | 0.200 |
|  |  |  |  |  |  |  |  |  |  |
| Bone mineral density |  |  |  |  |  |  |  |  |  |
| Whole body (g∙cm^-2^) | 0.981 | 1.384 | 1.155 | 0.013 | 1.128 | 1.182 | 0.090 | 0.054 | 0.200 |
| Subhead (g∙cm^-2^) | 0.872 | 1.281 | 1.034 | 0.013 | 1.009 | 1.060 | 0.086 | 0.119 | 0.102 |
| Trunk (g∙cm^-2^) | 0.824 | 1.245 | 0.994 | 0.013 | 0.967 | 1.021 | 0.090 | 0.131 | 0.048 |
| Upper limbs (g∙cm^-2^) | 0.683 | 0.967 | 0.807 | 0.009 | 0.790 | 0.824 | 0.058 | 0.117 | 0.134 |
| Lower limbs (g∙cm^-2^) | 0.882 | 1.489 | 1.200 | 0.018 | 1.165 | 1.236 | 0.121 | 0.067 | 0.200 |
|  |  |  |  |  |  |  |  |  |  |
| Lean soft tissue |  |  |  |  |  |  |  |  |  |
| Whole body (kg) | 31.0 | 44.3 | 38.7 | 0.5 | 37.7 | 39.6 | 3.2 | 0.084 | 0.200 |
| Trunk (kg) | 14.6 | 21.5 | 18.2 | 0.3 | 17.7 | 18.7 | 1.7 | 0.082 | 0.200 |
| Upper limbs (kg) | 2.8 | 5.7 | 4.2 | 0.9 | 4.1 | 4.4 | 0.6 | 0.083 | 0.200 |
| Lower limbs (kg) | 10.2 | 16.0 | 13.2 | 0.2 | 12.8 | 13.6 | 1.3 | 0.118 | 0.122 |
|  |  |  |  |  |  |  |  |  |  |
| Fat tissue |  |  |  |  |  |  |  |  |  |
| Whole body (kg) | 4.7 | 40.9 | 15.4 | 1.0 | 13.5 | 17.4 | 6.5 | 0.155 | 0.007 |
| Trunk (kg) | 2.4 | 19.8 | 7.5 | 0.5 | 6.5 | 8.5 | 3.4 | 0.173 | 0.001 |
| Upper limbs (kg) | 0.2 | 4.5 | 1.3 | 0.1 | 1.0 | 1.5 | 0.9 | 0.150 | 0.011 |
| Lower limbs (kg) | 1.8 | 15.9 | 7.5 | 0.5 | 6.4 | 8.5 | 3.5 | 0.148 | 0.013 |
|  |  |  |  |  |  |  |  |  |  |

*DXA* dual energy x-ray absorptiometry, *SEM* standard error of the mean, *95% CL* 95% confidence limits
